# Clinical outcome of arthroscopic capsular release for frozen shoulder: essential technical points in 255 patients

**DOI:** 10.1186/s13018-018-0758-5

**Published:** 2018-03-16

**Authors:** Katsuaki Kanbe

**Affiliations:** Department of Orthopaedic Surgery, Tokyo Women’s Medical University, Medical Center East, 2-1-10 Nishiogu, Arakawa, Tokyo, Japan

**Keywords:** Frozen shoulder, Arthroscopic capsular release, LHB, CH ligament

## Abstract

**Background:**

The purpose of this study was to investigate the long-term clinical outcome and its related factors regarding the severity of adhesion of CH ligament over long head of biceps (LHB) after shoulder arthroscopic capsular release for frozen shoulder with technical points in 255 patients.

**Methods:**

We performed arthroscopic capsular release for frozen shoulder in 267 shoulders of 255 patients, 112 males and 143 females, with mean age of 56.39 years, mean disease duration periods of 0.934 years for conservative treatment, and mean follow-up periods of 5.6 years. The frozen shoulders were divided based on the severity of adhesion between CH ligament over LHB: those with slight degree of synovitis, no adhesion by obtuse rod, and slight thickness of the released capsule (type A), those with moderate degree of synovitis, moderate adhesion of the LHB by obtuse rod, and moderate thickness of the released capsule (type B), and those with severe degree of synovitis, severe adhesion of the LHB by obtuse rod, and severe thickness of the released capsule adhesion and a flatly shaped LHB (type C). We assessed the clinical factors related to the scoring of the shoulders by the criteria of the American Shoulder and Elbow Surgeons (ASES) and the relationship with severity of LHB adhesion.

**Results:**

The ASES scores improved at 5 years postoperatively in all three groups significantly. The range of motion also significantly improved in all three groups significantly. The severity of the LHB adhesion over the CH ligament was confirmed to influence the ASES scores before and after the arthroscopic capsular release. There was a significant difference between type A and type B (*p* < 0.0001) or type C (*p* < 0.0001) before and after surgery. Logistic regression analysis showed disease duration, diabetes mellitus (DM), and ASES score were significantly associated to the severity type of LHB, especially DM has high odds ratio and was a risk factor for LHB adhesion. There is no adverse event including dislocation or axillary nerve injury and recurrence after arthroscopic capsular release at 5 years after surgery.

**Conclusions:**

The long-term results of arthroscopic capsular release in frozen shoulder were confirmed in 255 patients. The severity of LHB adhesion over the CH ligament, a pathological condition related to DM as a risk factor, seems to play an important role in the functional outcome. Therefore, the sufficient release of LHB was essential technical point for arthroscopic capsular release in frozen shoulder.

## Background

While physiotherapy, analgesics for pain, steroid injection, and silent manipulation can all be effective for frozen shoulder, there has been no description of a long term with more than 200 patients of arthroscopic capsular release for frozen shoulder so far. It is reported recently that arthroscopic capsular release for frozen shoulder is effective and safe in several literatures [1–3]. Walther et al. reported that arthroscopic capsular release should be recommended as the early choice for treatment in persistent frozen shoulder in 54 patients [1]. On the other hand, Neviaser used the term “adhesive capsulitis” to reflect his findings in surgery [4]. In pathological aspect, the thickness of the coracohumeral (CH) ligament over 4 mm and joint capsule over 7 mm by MRI was important to the diagnosis of frozen shoulder [5]. In anatomical analysis, the CH ligament was divided into two parts: one part spread fibers over the rotator interval to the posterior portion of the greater tuberosity and the other part enveloped the superior portion of the subscapularis, supraspinatus, and infraspinatus tendons. The anterior CH ligament holds the subscapularis muscle and anchors the muscle to the coracoid process in a similar manner to that of the posterior coracohumeral ligament (CHL) enveloping the supraspinatus and infraspinatus over the long head of biceps (LHB) tendon [6]. We previously reported the classification of arthroscopic findings for frozen shoulder based on the LHB adhesion over CH ligament in 87 patients [7]. The hypothesis in this study is that LHB adhesion to CH ligament is associated with the long-term outcome of arthroscopic capsular release in frozen shoulder. The purpose of this study was to investigate the long-term clinical outcome in 255 patients and extract clinical factors related to the efficacy of shoulder arthroscopic capsular release for frozen shoulder.

## Methods

### Study design

**Fig. 1:**
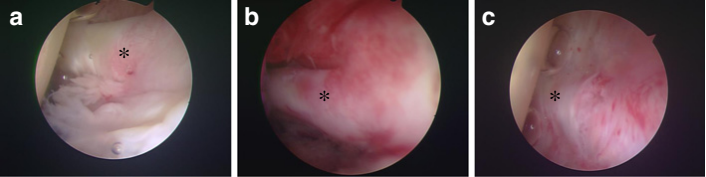
Arthroscopic classification based on the severity of adhesion of LHB and CH ligament. **a** Slight adhesion to easily get into the back of LHB by obtuse rod. **b** Moderate adhesion to hardly get into the back of LHB by obtuse rod. **c** Severe adhesion with no space to get into the back of LHB by obtuse rod. Asterisk is LHB

Two hundred and sixty seven consecutive frozen shoulders of 255 patients underwent arthroscopic capsular release admitted in Tokyo Women’s Medical University, Medical Center East by a single surgeon (K.K.) from August 2003 including 112 males and 143 females, with mean age of 56.39 ± 10.24, mean disease duration periods 0.934 ± 0.393 years, and mean follow-up periods 5.648 ± 4.060 (range 5–13) years (Table 1). Preoperative treatments for the frozen shoulder included rehabilitation or steroid or hyaluronic acid injections or non-steroid anti-inflammatory drugs (NSAIDs) before arthroscopic capsular release at least more than 6 months. The criteria for inclusion in this study were severe night pain concomitant with no improvement of flexion (90°) and external rotation (0°) and poor responsiveness to rehabilitation for at least 5 to 6 months prior to the surgery recognized on the thickness of CH ligament by MRI [5]. Exclusion criteria were complete rotator cuff tear, acromioclavicular subluxation, and biceps tendon rupture in clinical and MRI findings. The frozen shoulders were divided into three types based on the severity of the adhesion of the LHB to the CH ligament as assessed by arthroscopy (Fig. 1): those with slight degree of synovitis, no adhesion by obtuse rod, and slight thickness of the released capsule (type A), those with moderate degree of synovitis, moderate adhesion of the LHB by obtuse rod, and moderate thickness of the released capsule (type B), and those with severe degree of synovitis, severe adhesion of the LHB by obtuse rod, and severe thickness of the released capsule adhesion and a flatly shaped LHB (type C). The frozen shoulders (*n* = 267) were divided into 162 shoulders of type A shoulders (56.20 ± 11.20 years; range, 23–82 years), 87 shoulders of type B shoulders (56.61 ± 8.06 years; range, 36–76 years), and 18 shoulders of type C shoulders (57.06 ± 11.13 years; range, 35–78 years). Disease duration with conservative treatment before surgery was 0.790 ± 0.271 years in type A, 1.075 ± 0.362 years in type B, 1.556 ± 0.591 years in type C.

**Table 1:** Baseline characteristics for arthroscopic capsular release

Patient/shoulder number	255/267
Age (years)	56.39 ± 10.24
Female (*n*/%)	143 (53.56)
Disease duration (years)	0.934 ± 0.393
Follow-up period (years)	5.648 ± 4.060
Type A (*n*/%)	162 (60.67)
Type B (*n*/%)	87 (32.58)
Type C (*n*/%)	18 (6.74)
ASES scores at baseline	41.104 ± 5.965
DM (*n*/%)	53 (19.85)

*ASES* American Shoulder and Elbow Surgeons, *DM* diabetes mellitus

### Procedure of arthroscopic capsular release and essential technical points for frozen shoulder: partial capsular release and ASD

**Fig. 2:**
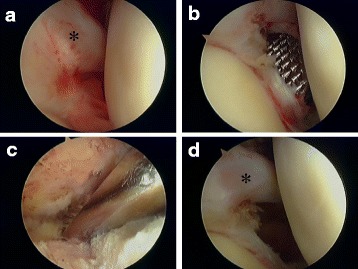
Procedure of arthroscopic capsular release for frozen shoulder. **a** Arthroscopic finding around LHB with synovium over CH ligament. Asterisk is LHB. **b** Rasp is used just outside of labrum along with the glenoid neck bone. **c** Subacromial decompression was performed concomitantly by using abrader arthroscopically. **d** After arthroscopic capsular release, CH ligament adhered over LHB was removed and joint space was widen clearly

After placing the patient in the beach-chair position under general anesthesia or interscalene local anesthetic blockade, the shoulder was examined before surgery to assess the range of motion in flexion and extension, external rotation at 0° abduction, external rotation at 90° abduction, and internal rotation at 90° abduction. After introducing a 4-mm arthroscopy through a standard posterior portal and performing an initial diagnostic arthroscopy, we created an anterior portal just lateral side of coracoid process to superior of the subscapularis tendon using the outside-in technique in order to facilitate maneuvers by instruments such as shavers and a radiofrequency instrument (VAPR®; Mitek, Norwood, MA). Next, we assessed the LHB adhered to the CH ligament over shoulder joint (Fig. 2a). Our first step in the capsular release was to eliminate the adhesion of the LHB to the CH ligament using a radiofrequency instrument. Next, we removed the joint capsule just next to the labrum using a radiofrequency instrument and rasp from 5 o’clock to 11 o’clock of the right-side shoulder over LHB (Fig. 2b). Our method is partial capsular release for frozen shoulder. Thus, we released the anterior, anteroinferior, superior, and superior-posterior capsules in addition to eliminate the LHB adhesion to the CH ligament. Inferior-posterior portion of capsule was remained to maintain shoulder stability and refrain from axillary nerve injury. A rasp conventionally used for arthroscopic Bankart repair proved quite useful in moving the capsule into the neck of the glenoid without axillary nerve complication to move the capsule. After arthroscopically observing the joint, we moved a scope into the subacromial space via a lateral and antero-lateral portal, shaved the synovium in the subacromial bursa, and carefully observed the rotator cuff. Arthroscopic subacromial decompression (ASD) was performed and smoothed the surface of rotator cuff and subacromial bursa by using VAPR® and the rasp (Fig. 2c). Then, after removing the scope, we performed the manipulation. Once the scope and instruments were removed, shoulders were manipulated in external rotation at 0° of abduction, external rotation at 90° of abduction, internal rotation at 90° of abduction, and flexion in the plane of the scapula in addition to extension. At the end of the capsular release, the measurement of range of motion obtained after the manipulation was performed. After all procedures, we checked the sliding movement of LHB and wash out intra GH joint to eliminate the coagulation and debris for final step (Fig. 2d). If the insufficient ROM was obtained, the adhesion of LHB should be released again.

As postoperative rehabilitation protocol, passive, assisted-active exercises and stooping exercise were commenced for forward flexion and external rotation 1 day after surgery with the assistance of a physical therapist. After 2 week of passive exercise, the patients began active exercise to strengthen the rotator cuff and scapular stabilizers. After the rehabilitation for 4 to 6 weeks, the patients were back on normal work schedules without any limitations to daily activity. The rehabilitation was still continued for 3 months after surgery to obtain complete muscle strength of the shoulder.

### Measurement of outcome

All patients were assessed by the American Shoulder and Elbow Surgeons (ASES) score preoperatively, and at the final evaluation was performed at an average of 5.648 ± 4.060 years postoperatively [8]. Preoperative and postoperative assessments for the progress of recovery of the range of motion at forward elevation (flexion), external rotation at 0 and 90° of abduction, and internal rotation at 0 and 90° of abduction were performed in the three arthroscopic types (types A, B, and C). Informed consent was obtained from all patients, and the study protocol was approved by the ethics committee of Tokyo Women’s Medical University. ASES scores were assessed in each three groups before and after surgery, and multiple regression analysis with logistic procedure was used for detecting the clinical factors related to the severity of LHB type. The population especially of diabetes mellitus (DM) in each group was analyzed.

### Statistical analysis

We used the Wilcoxon test to compare ASES scores [8] and the degrees of range of motion with before and after surgery. Mann-Whitney *U* test was used to compare those results among different types of groups. The logistic regression analysis for LHB type severity was performed including age, disease duration, DM, and ASES scores at baseline and 5 years after surgery. Gender ratio was also calculated in each group. *p* values < 0.05 were considered to be significant using StatFlex version 6.0 (Statflex, Tokyo, Japan).

## Results

**Fig. 3:**
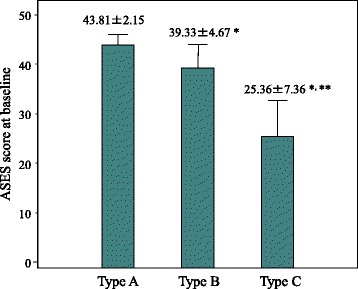
ASES scores at baseline in each group. Asterisk indicates significant difference compared with type A (*p* < 0.001). Two asterisks indicate significant difference compared with type B (*p* < 0.001)

**Fig. 4:**
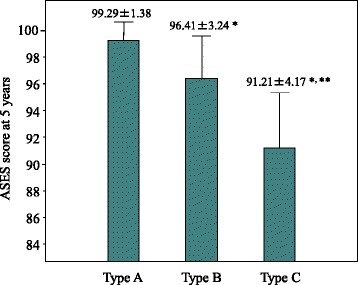
ASES scores at 5 years after arthroscopic capsular release in each group. Asterisk indicates significantly different compared with type A (*p* < 0.001). Two asterisks indicate significantly different compared with type B (*p* < 0.001)

**Fig. 5:**
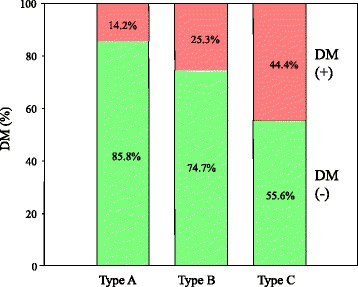
The ratio of the patients with DM in each group. DM ratio of type C was significantly higher than that of type A (*p* = 0.0012) and type B (*p* = 0.0302)

The ASES score improved postoperatively in all three groups: from 41.10 ± 5.96 before surgery to 97.81 ± 3.25 at 5 years after surgery in the 267 shoulder joints, including from 43.81 ± 2.15 before surgery to 99.29 ± 1.38 after surgery in the type A shoulder joints (*n* = 162), from 39.33 ± 4.67 to 96.41 ± 3.24 in type B (*n* = 87), and from 25.36 ± 7.36 to 91.21 ± 4.17 in type C (*n* = 18) (Figs. 3 and 4). There was a significant difference between type A and type B (*p* < 0.0001) or type C (p < 0.0001) before and after surgery. The range of motion in flexion improved in all three groups postoperatively, from a mean of 80 ± 6.11 to 165 ± 8.84 in type A, from a mean of 75 ± 5.58 to 155 ± 7.96 in type B, and from a mean of 60 ± 6.38 to 140 ± 7.55 in type C. External rotation at 0° of abduction was improved from a mean of − 10 ± 7.32 to 45 ± 6.51 in type A, from a mean of − 15 ± 7.11 to 40 ± 6.89 in type B, and from a mean of − 25 ± 6.98 to 30 ± 7.45 in type C. Internal rotation improved from a mean of S1 to Th12 in type A, from a mean of S2 to L1 in type B, and from a mean of S2 to L1 in type C. Therefore, the range of motion was also confirmed to be dependent on the recovery of LHB adhesion to the CH ligament after surgery. Logistic regression analysis revealed the arthroscopic finding as for type of LHB adhesion related with disease duration (*p* = 0.0012, odds ratio 0.08723, RI 0.02004~0.37964), DM (*p* = 0.0005, odds ratio 6.96680, RI 2.34963~20.6570), ASES score at baseline (*p* < 0.0001, odds ratio 1.56785, RI 1.29615~1.89651), and ASES scores at 5 years (*p* = 0.0014, odds ratio 1.60086, RI 1.19857~2.13819) (Table 2). Furthermore, the percent of DM in each group showed 14.2% in type A, 25.3% in type B, and 44.4% in type C as shown in Fig. 5. DM ratio of type C was significantly higher than that of type A (*p* = 0.0012) and type B (*p* = 0.0302). Female percent was 44.4% in type A, 65.5% in type B, and 77.8% in type C. Female ratio of type C was significantly higher than that of type A (*p* = 0.0070) and type B (p = 0.0014). However, logistic analysis showed no significant difference to the type of LHB (*p* = 0.0974). Therefore, LHB adhesion to the CH ligament related to clinical outcome and DM ratio in frozen shoulder. There was no adverse event including axillary nerve injury or dislocation and recurrence after arthroscopic capsular release in this study.

**Table 2:** Logistic regression analysis for the type of frozen shoulder

Factors	*p* value	Odds ratio (RI)
Age	0.8394	1.00381 (0.96759~1.04137)
Disease duration	0.0012	0.08723 (0.02004~0.37964)
DM	0.0005	6.96680 (2.34963~20.6570)
ASES score at baseline	< 0.0001	1.56785 (1.29615~1.89651)
ASES score at 5 years	0.0014	1.60086 (1.19857~2.13819)
Gender	0.0974	1.93661 (0.88640~4.23110)

*ASES* American Shoulder and Elbow Surgeons, *DM* diabetes mellitus

## Discussion

Management of choice involves conservative treatment such as non-steroidal anti-inflammatory drugs (NSAIDs), intra-articular steroids of hyaluronic acid injection, physical therapy, and silent manipulation under cervical nerve root block anesthesia are applied [9–12]. However, Cochrane reviews have demonstrated that the current literature base shows that physiotherapy alone has little to no benefit as compared to control groups [13]. There are a number of adjuncts that are often used with physiotherapy including extracorporeal shockwave therapy, electromagnetic stimulation, acupuncture, and the use of lasers, none of which have been subjected to investigation with randomized controlled studies [14]. Even when undergoing rehabilitative treatment, frozen shoulder often continues to feel severe night pain and contracture enough to disturb shoulder function. In some 10% of cases, indications for arthroscopic capsular release are present and currently, shoulder arthroscopic capsular release is a treatment of choice in such cases [14]. We selected arthroscopic capsular release for recalcitrant adhesive frozen shoulder after unsuccessful rehabilitation. However, the comparison of manipulation and arthroscopic capsular release by systemic review was reported that the quality of evidence available is low and the data available demonstrate little benefit for a capsular release instead of, or in addition to, a manipulation under anesthesia [15]. Ogilvie-Harris et al. attempted to compare manipulation with arthroscopic release on a prospective cohort of 40 patients [16]. The release induced removal of synovium from the rotator interval, release of the anterior glenohumeral ligament and the intra-articular portion of the subscapularis tendon, and finally, division of the anterior half of the inferior capsule. Their results after a follow-up of between 2 and 5 years showed a similar range of movement, but the release had a much better outcome in review literature [17]. However, there was no evidence of the efficacy of arthroscopic capsular release in more than 200 patients in long-term results.

Our first observation in the current investigation was the restriction of dynamic sliding movement of the LHB in frozen shoulder compared with the normal [7]. The LHB stands upward from the IR to ER positions during this movement. The mechanical physiological functions of the shoulder depend quite closely and sensitively on this area of the LHB, especially for ER. After arthroscopic capsular release, the ER improved in the patients who exhibited the dynamic sliding movement of the LHB. Our data indicated that the physiological movement of the LHB to the rotator interval plays a key role in acquiring an improved range of motion in shoulders rated with high ASES scores. Furthermore, MRI findings on frozen shoulder have typically revealed a thickening of the coracohumeral ligament (CHL) [5]. CHL thickness and wide spread was evident in all three types especially in type C.

Frozen shoulder is thought to have an incidence of 3–5% in the general population and up to 20% in those with diabetes [18]. Its peak incidence in between the ages of 40 and 60 is rare outside these age groups and in manual workers [19] and is slightly more common in women. In this study, DM ratio was 19.85% in total cases. Experimental analysis for frozen shoulder, we reported that mechanical stress on the LHB and rotator interval (RI) in the shoulder may induce the tissue around LHB of mitogen-activated protein (MAP) kinases to express nuclear factor (NF)-κB by CD29 in order to develop capsule contracture, producing matrix metalloproteinase (MMP)-3, interleukin(IL)-6, and vascular endothelial growth factor (VEGF) [20]. Therefore, vascularity of capsule in frozen shoulder was evident in arthroscopic finding. DM also expressed those molecule to induce fibrous tissue in the area of the mechanical stress such as CH ligament and LHB. DM was found to be a possible risk factor related to the severity LHB adhesion with CH ligament which was wide spread out abnormally. Therefore, the patient of frozen shoulder with DM should be careful to manage the arthroscopic capsular release especially around LHB.

In technical point of view, the superior release is then extended to reach the long head of biceps and is continued to release the CHL in the plane between the superior glenoid and supraspinatus. If internal rotation or adduction of the shoulder is significantly restricted then the camera portal can be reversed to anterior portal for a posterior capsular release. Some surgeons complete the inferior release with a gentle manipulation but some surgeons advocate a full 360° capsulectomy under direct vision while accepting the higher risk of iatrogenic injury the axillary nerve [21]. Pearsall et al. performed arthroscopic release of the anteroinferior capsule, the intra-articular portion of the tendon of subscapularis, the superior and middle gleno-humeral ligaments, and the coracohumeral ligament in patients who had been recalcitrant to conservative treatment [22]. Among the 35 patients followed at a mean of 22 months after surgery, 83% had normal or only mildly symptomatic shoulders. These patients also received a tapered 21-day course of oral prednisolone. None of our patients were given oral steroids during the treatment. We consider that 1 month period is the most important window for obtaining better results by rehabilitation after arthroscopic capsular release. Most patients obtain their final range of motion by 4 to 6 weeks after capsular release. We released the anterior, antero-inferior, and superior capsules in addition to eliminating the LHB adhesion to CHL. Detailed arthroscopy assessments of the LHB adhesion revealed the clinical mechanism responsible for the decreased shoulder function associated with frozen shoulder. Limitation of study includes no control study and more long results needed to the recurrence of this procedure, and the mechanism of DM which contributed the severity of adhesion over LHB was still unclear. We found the risk factor of clinical outcome was DM condition. Therefore, it is possible to DM frozen shoulder should be separated to another category compare to idiopathic frozen shoulder in pathologic condition. In the future, arthroscopic capsular release with less pain after surgery should be performed in day surgery for the privilege of the patients with frozen shoulder.

## Conclusions

The long-term results of arthroscopic capsular release in frozen shoulder were confirmed in 255 patients. The severity of LHB adhesion over the CH ligament, a pathological condition related to DM as a risk factor, seems to play an important role in the functional outcome. Therefore, the release of LHB was essential technical point for arthroscopic capsular release in frozen shoulder.

## Abbreviations

ASES: American Shoulder and Elbow Surgeons
CH: Coracohumeral
DM: Diabetes mellitus
LHB: Long head of biceps
